# The Popular Science Monthly

**Published:** 1873-04

**Authors:** 


*The Popular Science Monthly.*

Published by D. Appleton & Co., New York. Price, five dollars a year; single numbers, fifty cents. The number for April is the concluding number of the first annual volume of this magazine, which is of the highest order of any magazine published in this country for general reading. It is a well-conducted organ of views of such men as Herbert Spencer, Dr. Draper, and Profs. Brewer, Henry and Le Conte. Exclusive of editorial matter and miscellany, the present issue contains the following articles: On the Importance of the Cultivation of Science, by Prof. Joseph Henry; The Nebular Hypothesis, by Prof. John Le Conte; River and Lake Terraces (illustrated); Applied Sanitary Science, by Dr. J. R. Black; Barberism in English Education, by Hon. E. E. White; The Horned Frog, by Frank Buckland (illustrated); On the Transfusion of Blood, by Gustav Lemattre; Science and our Educational System, by F. A. P. Barnard; The Troglodytes, or Cave Dwellers of France, by Paul Broca (illustrated); The Study of Sociology, by Herbert Spencer; English and American Science, by Herbert Spencer; English and American Science, by Prof. John W. Draper; Science and Public Affairs, by President Andrew E. White; Discovery of Mount Tyndall, by Prof. Wm. H. Brewer.

